# A cross-reactive mouse monoclonal antibody against rhinovirus mediates phagocytosis *in vitro*

**DOI:** 10.1038/s41598-020-66600-x

**Published:** 2020-06-16

**Authors:** Mohammad Amin Behzadi, Angela Choi, James Duehr, Roya Feyznezhad, Chitra Upadhyay, Michael Schotsaert, Peter Palese, Raffael Nachbagauer

**Affiliations:** 1 Department of Microbiology, Icahn School of Medicine at Mount Sinai, New York, NY USA; 2 Global Health and Emerging Pathogens Institute, Division of Infectious Diseases, Icahn School of Medicine at Mount Sinai, New York, NY USA; 3 Graduate School of Biomedical Sciences, Icahn School of Medicine at Mount Sinai, New York, NY USA; 4 Present Address: University of Pittsburgh School of Medicine, Pittsburgh, PA USA; 5 Department of Medicine, Division of Infectious Diseases, Icahn School of Medicine at Mount Sinai, New York, NY USA

**Keywords:** Antiviral agents, Microbiology, Virology, Viral infection

## Abstract

Rhinoviruses (RVs) are the main cause of the common cold worldwide. To date, more than 160 types of the virus have been recognized, categorized into three major species - A, B, and C. There are currently no approved vaccines available to prevent infection with RVs. To elicit antibodies against conserved regions located on capsid proteins of RV A viruses, mice were sequentially vaccinated with DNA plasmids encoding capsid proteins of different RV A types. After a final boost with whole virus, antibody-expressing hybridomas were generated. After isotyping, 11 monoclonal antibodies (mAbs) expressing an IgG subtype Fc-domain were selected for further expansion and purification. Three mAbs showed cross-reactivity against multiple strains of RV A viruses by ELISA, including strains A1A, A1B, A15, A16 and A49. Other mAbs had strain-specific binding patterns, with the majority of mAbs showing reactivity to RV-A15, the strain used for the final vaccination. We found that the RV-A15-specific mAbs, but not the cross-reactive mAbs, had neutralizing activity against RV-A15. An antibody dependent cellular phagocytosis (ADCP) assay revealed substantial ADCP activity for one of the cross-reactive mAbs. Epitope mapping of the neutralizing mAbs via escape mutant virus generation revealed a shared binding epitope on VP1 of RV-A15 for several neutralizing mAbs. The epitope of the ADCP-active, non-neutralizing mAb was determined by microarray analysis of peptides generated from the VP1 capsid protein. VP1-specific, cross-reactive antibodies, especially those with ADCP activity, could contribute to protection against RV infections.

## Introduction

Rhinoviruses (RVs) belong to the family of *Picornaviridae* and are known as a leading cause of respiratory infections. These viruses can also cause acute exacerbations of asthma and chronic obstructive pulmonary disease (COPD)^1,2^. Despite considerable efforts in past decades, no vaccines or therapeutics have yet been approved to combat RV infection^3,4^. The major obstacles in RV vaccine development are the large number of types and the lack of an appropriate animal model for preclinical evaluation of vaccine candidates^5–7^.

Currently more than 160 types of RVs have been identified^8^. Based on genetic diversity and phylogenetic sequence analysis, these types are classified into three species: RV A, B, and C^9^. So far, three different cellular membrane glycoproteins have been recognized as binding receptors for RVs. These include the intercellular adhesion molecule 1 (ICAM-1, used by the majority of RV A, and all RV B types), the low-density lipoprotein receptor family members (LDLR, used by the minority of RV A types), and the cadherin-related family member 3 (CDHR3; used by RV C)^10^. The genomic RNA of RVs is surrounded by an icosahedral capsid shell that consists of 60 copies of four proteins: VP1, VP2, VP3, and VP4. The outer surface of this capsid is made up of VP1, VP2, and VP3, whereas VP4 is localized internally at the interface between the capsid and the viral genome^11^. These three exterior capsid proteins form a canyon structure that allows RV viruses which bind to ICAM-1 to engage their receptor on the surface of target host cells^12–14^.

Antibodies raised against the capsid proteins of RVs are the primary host defense against RV infection^15^. VP1 is the most exposed surface protein, and plays a critical role in viral antigenicity and induction of neutralizing antibodies^16^. Although neutralizing antibodies elicited by infection can reduce viral replication, only limited cross-protection against heterologous strains is provided because of the large antigenic diversity of RVs^17^. Previous attempts to establish cross-type protection using vaccines containing multiple conserved regions of the virus had some success in eliciting neutralizing responses^18–21^. Despite these early successes, whether or not viable cross-reactive targets for cross-protective vaccines exist remains an open question.

To further identify potential future vaccination target epitopes, we utilized a sequential immunization strategy in mice with heterologous RV A antigens. In this study, we identified three cross-reactive monoclonal antibodies (mAbs). While these mAbs did not exhibit neutralizing activity, one mAb interestingly showed a high level of activity in an antibody-dependent cellular phagocytosis (ADCP) assay.

## Results

### Hybridoma generation and screening

**Figure 1:**
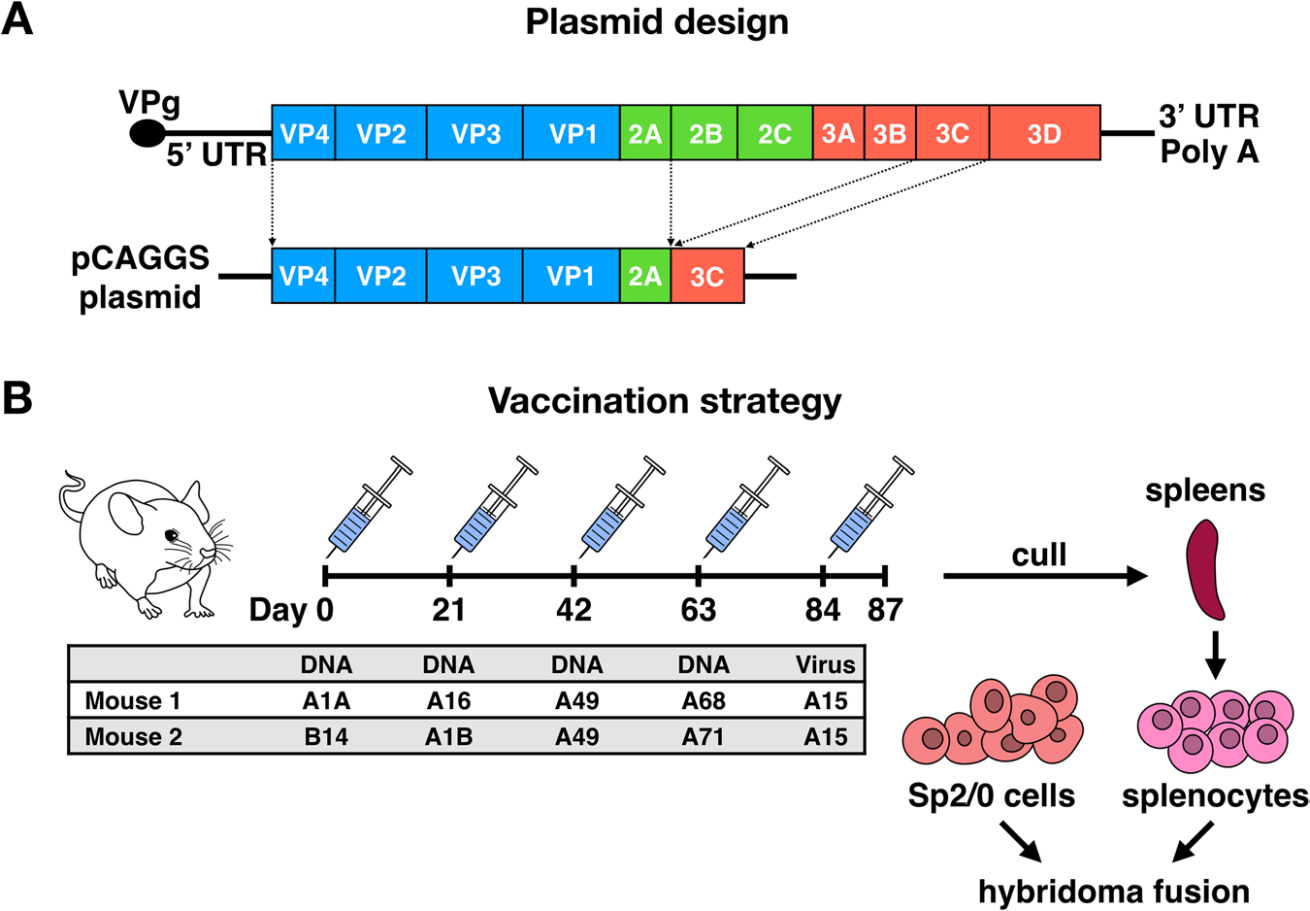
(**A**) Schematic representation of RV DNA vaccine constructs (bottom). Genome structure of RV (top). The recombinant pCAGGS plasmids encode four capsid proteins (VP1-4), and two proteases (2A and 3C) of RVs. (**B**). Mouse immunization strategy. Mice received four vaccinations with 75 µg of recombinant DNA constructs at 3-week intervals followed by a final boost with 50 μg of purified RV-A15. Three days after the final boost, the animals were euthanized; the spleens were harvested, and used for hybridoma fusion.

**Figure 2:**
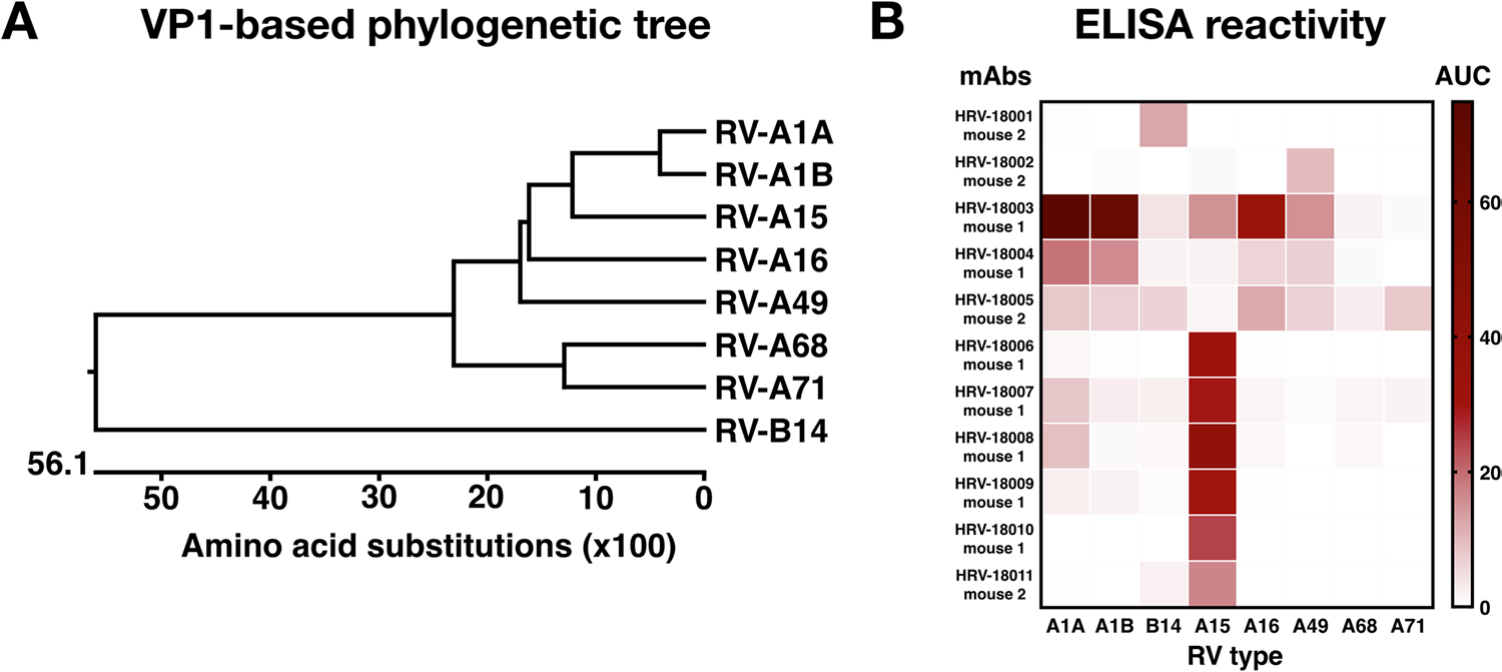
(**A**) Phylogenetic tree of RVs based on VP1 amino acid sequences. (**B**) Reactivity of isolated mAbs against different types of RV. The heat map represents the area under the curve (AUC) of ELISAs against different types of RV. Three mAbs (HRV-18003, HRV-18004, and HRV-18005) showed cross-reactivity against multiple types of RV by ELISA, including A1A, A1B, A15, A16 and A49. Additional mAbs showed strain-specific binding patterns, with the majority showing reactivity to RV-A15, the virus used for the final vaccination.

To generate hybridomas, two female BALB/c mice were sequentially vaccinated with recombinant pCAGGS plasmids encoding diverse capsid proteins and two proteases of RVs, to facilitate proper protein cleavage (Fig. 1A). The immunizations were followed by a final boost with purified whole virus using the RV-A15 strain (Fig. 1B). Each round of vaccination was performed with plasmids encoding for a single type of RV as illustrated in the table in Fig. 1B. Since RVs cannot bind murine ICAM-1, intramuscular injection of RV-A15 was not expected to result in virus replication, but solely to provide an antigenic stimulus. Hybridomas were screened for RV reactivity and Ig isotypes. Eleven IgG mAbs were selected for further characterization based on robust reactivity against at least one of the RV types used during the immunization regimen. Of these mAbs, 3 showed cross-reactivity against multiple types of RV A viruses by ELISA, including A1A, A1B, A15, A16 and A49 (Fig. 2A,B). Additional mAbs showed strain-specific binding patterns, with the majority showing reactivity to RV-A15, the virus used for the final vaccination (Fig. 2B, Supplementary Fig. 1).

### Strain-specific, but not cross-reactive antibodies, show neutralizing activity

**Figure 3:**
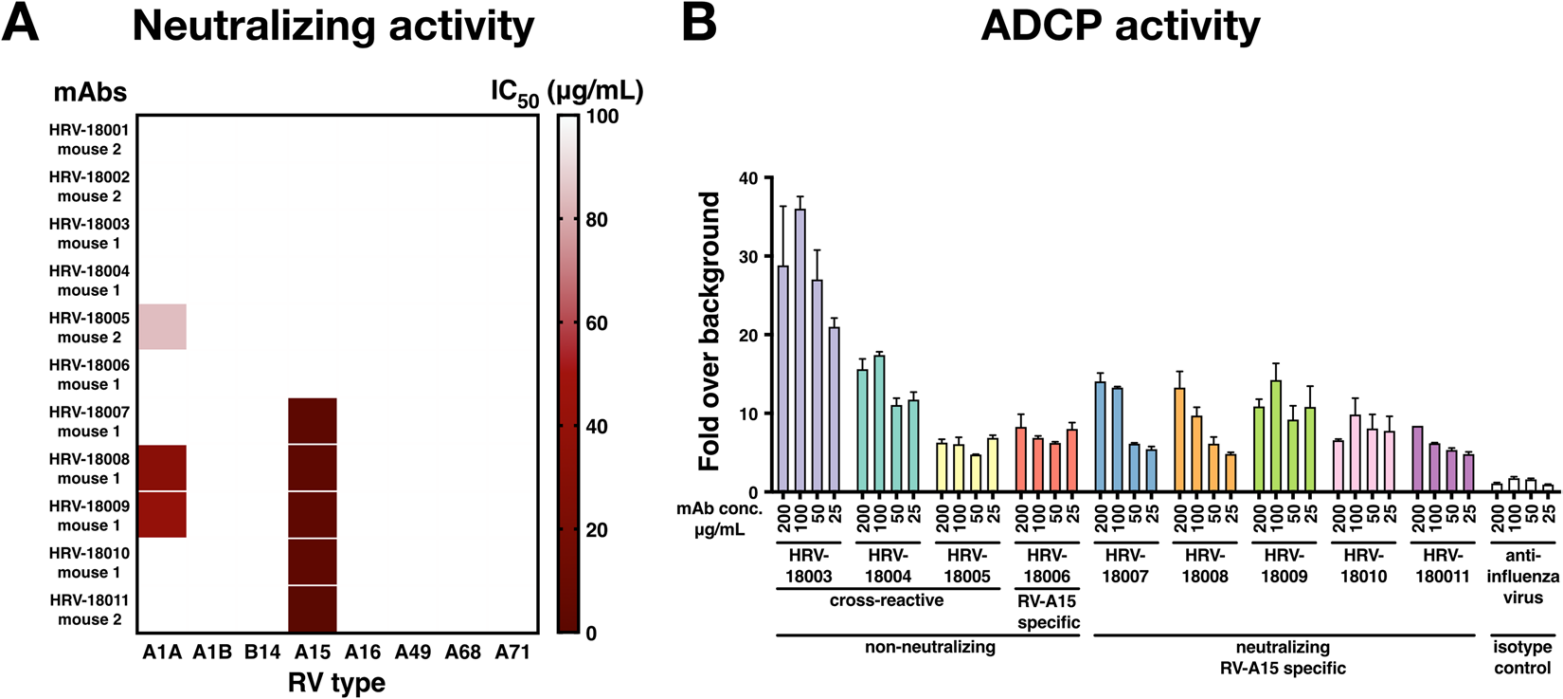
(**A**) Neutralizing activity of mAbs against multiple types of RV. The heat map shows the IC_50_ values for neutralizing activity of each tested mAb against different types of RV. Most of the RV-A15-specific mAbs, but not the cross-reactive mAbs, were highly neutralizing. The IC_50_ values were calculated by fitting data with a four-parameter nonlinear regression curve using GraphPad Prism. (**B**) Antibody dependent cellular phagocytosis (ADCP) activity of mAbs against RV-A15. Using a flow-cytometry based assay, one of the cross-reactive mAbs, mAb HRV-18003, elicited a high level of ADCP activity. An anti-influenza virus mAb (2B9; IgG2a) was used as a negative control. Values are depicted as fold induction over cells and virus-conjugated beads without antibody. All assays were performed in duplicate. Bars show the mean and error bars indicate the standard error of the mean.

To characterize the functionality of these mAbs, we first explored their neutralizing activity against a panel of RV types. Using a microneutralization assay, we found that five RV-A15-specific mAbs were highly neutralizing, with IC_50_ values ranging from 0.827 μg/ml for mAb HRV-18011 to 2.638 μg/ml for mAb HRV-18009. Interestingly, none of the cross-reactive mAbs (HRV-18003, HRV-18004, and HRV-18005), showed neutralizing activity (Fig. 3A, Supplementary Fig. 2).

### A cross-reactive mAb shows potent ADCP activity

In addition to neutralizing activity, Fc-mediated effector functions are becoming an important topic for antiviral immunity. Therefore, we investigated the capacity of the RV-A15-reactive mAbs to induce ADCP activity in a mouse macrophage cell line *in vitro* (Supplementary Fig. 3). This flow-cytometry based ADCP assay revealed a high degree of ADCP activity for mAb HRV-18003 (on the order of ~30-fold signal over background), one of the cross-reactive mAbs we identified (Fig. 3B). The other two cross-reactive mAbs, HRV-18004 and HRV-18005, scored markedly lower in ADCP activity (~10-fold and 8-fold signal over background, respectively). Among the RV-A15-specific neutralizing mAbs, HRV-18009 appears to have the highest ADCP activity (~10-fold signal over background). We observed a similarly strong ADCP activity for HRV-18003 against RV-A1A and RV-A16 (Supplementary Fig. 4).

### RV-A15-neutralizing mAbs bind a shared epitope on VP1

**Figure 4:**
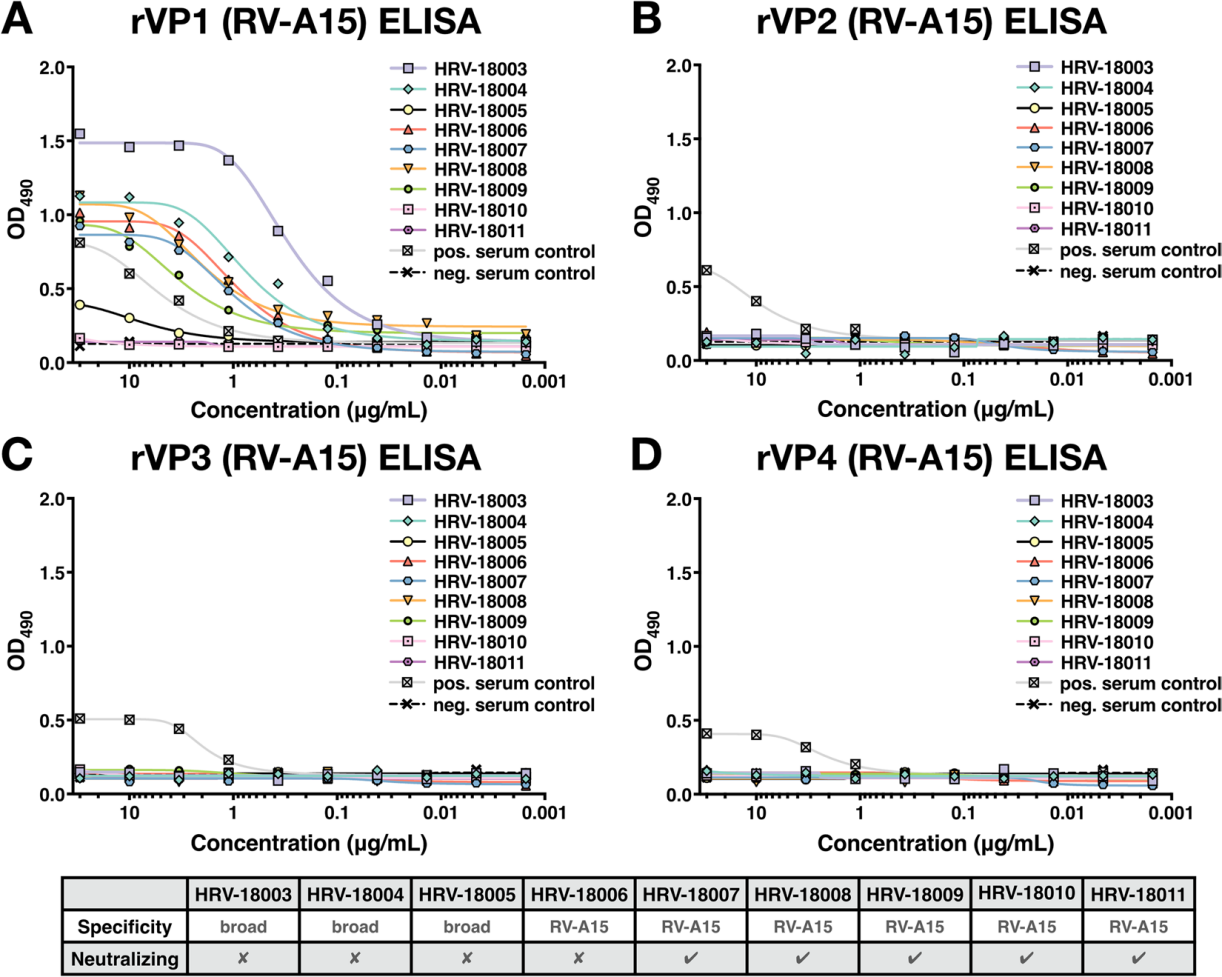
Capsid protein specificity. The reactivity of 6 RV-A15-specific mAbs and 3 cross-reactive mAbs was tested against recombinantly expressed VP1 **(A)**, VP2 **(B)**, VP3 **(C)** and VP4 **(D)** capsid proteins of RV-A15 using ELISA. The table summarizes the specificity (breadth of binding against multiple types) and neutralizing activity of each mAb.

To identify the targets of the RV-A15-specific mAbs, the 6 RV-A15-specific and 3 cross-reactive mAbs were tested against recombinantly expressed VP1-VP4 capsid proteins of RV-A15 (Fig. 4). The ELISA results showed that 2 cross-reactive mAbs and 4 RV-A15-specific, neutralizing mAbs bound to the recombinant VP1 protein. Only weak binding was observed for cross-reactive mAb HRV-18005, while the other two RV-A15-specific mAbs did not bind to any of the recombinant proteins, potentially indicating binding sites that span across multiple proteins.

**Figure 5:**
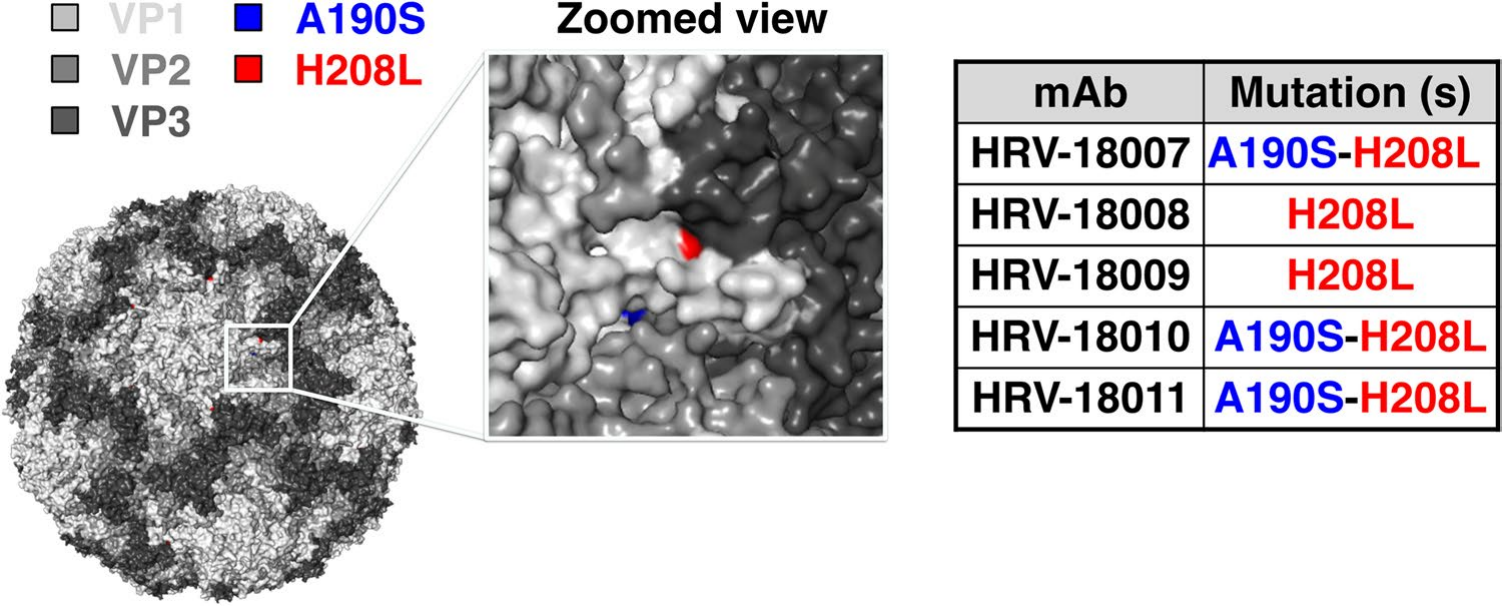
Binding site of RV-A15-neutralizing mAbs. Escape mutations are visualized on a published crystal structure of RV-A1A (PDB accession no. 1R1A). The two critical residues, A190S and H208L, were highlighted in blue and red, respectively, as visualized in PyMoL (Schrödinger, version 2.3.2, www.pymol.org).

The residues critical for RV-A15-neutralizing mAbs binding were identified through the generation of escape mutant viruses. After serial passage in the presence of mAb and subsequent sequencing, two critical residues were identified for mAbs HRV-18007, HRV-18010, and HRV-18011. The mutations were observed on the βG strand and the GH loop of VP1 of RV-A15 (A190S-H208L). Only the H208L residue changed in the escape mutants for mAbs HRV-18008 and HRV-18009 (Fig. 5). In order to confirm that the escape mutants had truly “escaped” mAb binding, microneutralization assays were performed on the five escape mutant viruses against all five mAbs. The results revealed no neutralization of escape mutant viruses by the tested mAbs, further confirming a shared binding epitope of the neutralizing mAbs on the VP1 capsid protein (Supplementary Fig. 5).

### Cross-reactive mAbs HRV-18003 and HRV-18004 bind a conserved epitope on VP1

We next aimed to characterize the binding of the cross-reactive mAbs. In addition to the ELISA against recombinant VP proteins, a reducing SDS-PAGE under denaturing conditions, followed by Western blotting was performed. Both mAbs HRV-18003 and HRV-18004 showed reactivity against a protein with the approximate size of 37 KDa for multiple RV types, consistent with the size of VP1 (Supplementary Fig. 6). The other cross-reactive mAb, HRV-18005, only showed reactivity to whole RV particles under non-denaturing conditions, suggesting that the epitope for that mAb was disrupted under denaturing conditions (data not shown).

**Figure 6:**
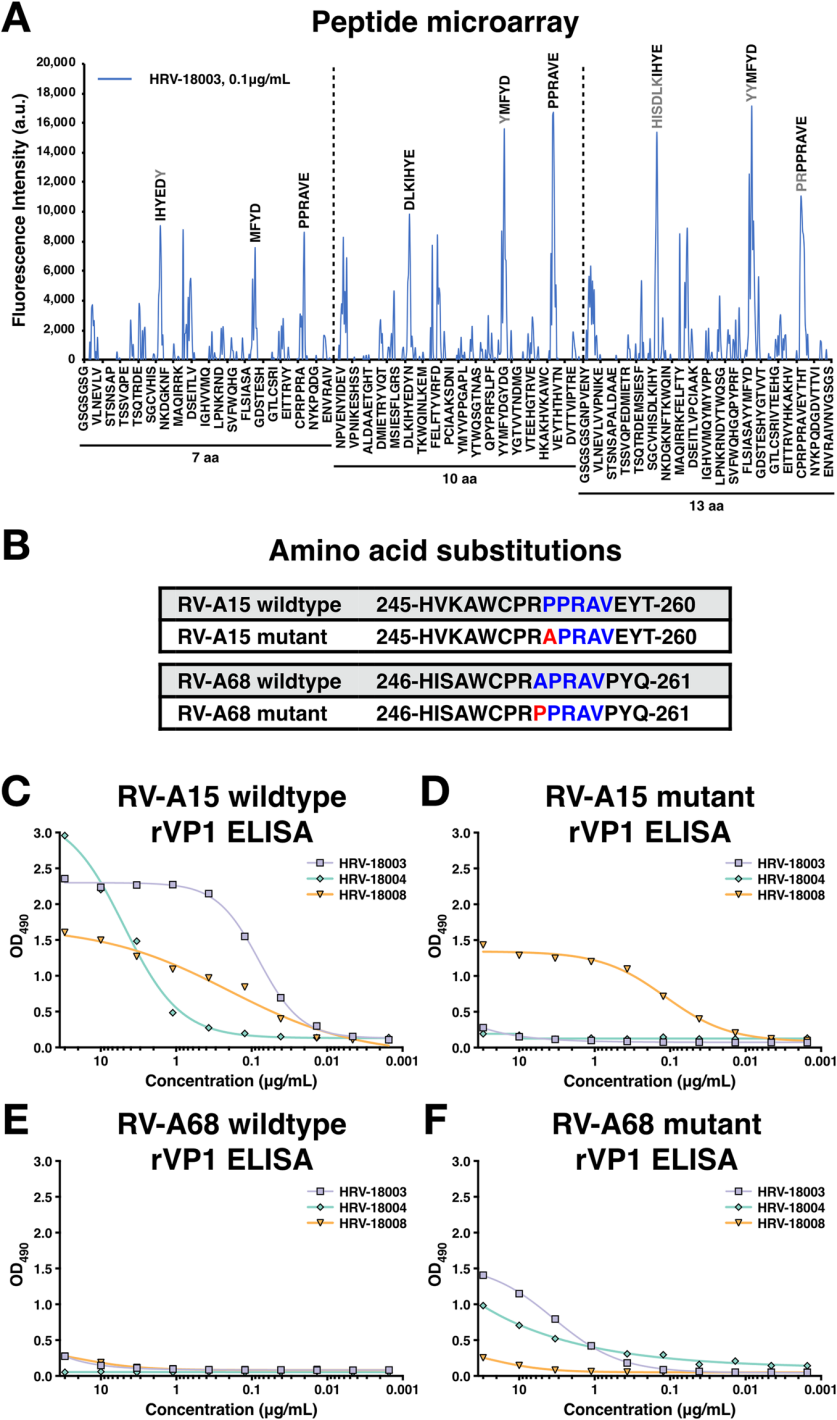
(**A**) Epitope mapping of cross-reactive mAb HRV-18003. Spot intensities were quantified following incubation of mAb HRV-18003 with overlapping cyclic peptides of 7, 10 and 13aa derived from VP1. **(B**) Amino acid sequences of recombinant RV-A15 and RV-A68 VP1 wildtype and mutant proteins. Highlighted in blue is the presumed epitope of mAb HRV-18003. Introduced single amino acid mutations are highlighted in red. **(C–F**) Reactivity of mAbs HRV-18003, HRV-18004, and HRV-18008 against recombinant VP1 proteins. Binding of mAbs by ELISA is shown against wildtype and mutant VP1 recombinant proteins of RV-A15 and RV-A68.

The epitope of the strongly ADCP-active, non-neutralizing mAb HRV-18003, was further investigated by a microarray analysis using overlapping cyclic peptides with lengths of 7, 10 or 13 amino acids (aa) derived from the VP1 capsid protein. A moderate to strong response profile at moderate signal-to-noise ratios was observed against peptides with the consensus motifs HISDLKIHYE, YYMFYD and PRPPRAVE (Fig. 6A). Despite showing similar peak intensities, visual assessment showed the clearest spot morphologies for peptides with the consensus motif PRPPRAVE, which was therefore regarded as the most likely epitope of mAb HRV-18003. The binding of peptides with the consensus motifs HISDLKIHYE and YYMFYD presumably resulted from non-specific interaction with multiple aromatic and acidic amino acids.

By aligning the VP1 sequences of RV-A15 (bound by mAb HRV-18003) and RV68 (not bound by mAb HRV-18003), we identified a single amino acid difference in the putative epitope PRPPRAVE (P253A). We therefore generated a mutant RV-A15 VP1 introducing the alanine of RV-A68. Further, we produced both a wildtype RV-A68 VP1 protein and a mutant protein in which the alanine was replaced with a proline (Fig. 6B).

We then tested binding of both cross-reactive mAbs HRV-18003 and HRV-18004 by ELISA against these recombinant proteins. Both mAbs recognized wildtype RV-A15 VP1, but lost binding to the RV-A15 mutant VP1. RV-A15-specific mAb HRV-18008 - which binds a different epitope as described above – showed binding against both wildtype and mutant VP1 of RV-A15 (Fig. 6C,D). Furthermore, when the point mutation was introduced into RV-A68 VP1, binding of mAbs HRV-18003 and HRV-18004 was observed (Fig. 6E,F). These data suggest that mAbs HRV-18003 and HRV-18004 both bind to the epitope PRPPRAVE on VP1. Interestingly, based on a mapping of the epitope on the structure of RV-A1A (utilizing PDB accession no. 1R1A), the P253A mutation is not exposed on the surface of the virion (Supplementary Fig. 7). This could indicate that the epitope is only exposed during virion “breathing”^22^, which could help explain the lack of neutralizing activity by the cross-reactive antibodies.

## Discussion

In the present study, we utilized a sequential DNA vaccination approach expressing the capsid proteins of heterologous types of RV, followed by a final whole virus boost with RV-A15 to elicit cross-reactive antibodies. Interestingly, a large proportion of mAbs isolated from the spleens of vaccinated mice were specific to the final vaccine strain, which was administered only 3 days prior to splenectomy. Four of the neutralizing RV-A15-specific mAbs were isolated from the same mouse and likely were clonally related, based on their binding of a shared epitope on VP1. However, mAb HRV-18011 was isolated from a different mouse, but bound the same epitope. Based on the rapid response, it is possible that the epitope is immunodominant in BALB/c mice.

We also isolated 3 cross-reactive mAbs that did not show neutralizing activity. Two of these mAbs bound a shared epitope on VP1, which was identified using a peptide microarray and further confirmed by testing binding to mutant recombinant VP1 proteins. Based on mapping of the identified epitope, it is not exposed on the virion surface. It is therefore possible that the mAbs can only bind to the epitope during viral “breathing”, which could expose epitopes otherwise buried within the capsid^23–26^. The third antibody did not bind well to the tested recombinant protein and showed binding by Western blot only under non-denaturing conditions, which could indicate binding to a conformational epitope spanning multiple proteins or to a non-capsid protein. Importantly, since these antibodies do not seem to interfere with viruses binding to the host cells, it is possible that their protective effect would be infection permissive, similar to Fc-mediated protection by antibodies against other viral infections^27^.

The binding of the cross-reactive antibodies did not span all types of RV A. It would be therefore important to identify additional epitopes that could broaden cross-reactive responses against all types of RV A. Since RV C viruses especially are a major cause of asthma, antibodies targeting similar conserved epitopes in these viruses could fill an important therapeutic gap. Interestingly, a recent editorial by Stepanova *et al*. identified a number of conserved epitopes (including epitopes in the C-terminal region of VP1, similar to the epitope bound by mAb HRV-18003) that could enable the generation of additional cross-reactive antibodies^28^. Further, systematic vaccination experiments focusing on specific RV clusters could reveal more detailed information on the cross-reactive potential of mAbs.

Importantly, one of the cross-reactive mAbs (HRV-18003) showed strong activity in an *in vitro* ADCP assay. Recent advances in understanding the mechanisms of antibody functions have revealed the critical importance of Fc-mediated effector activities such as ADCP and ADCC in clearance of virus and virus-infected cells^29,30^. ADCC would likely not contribute to protection against RV, since the capsid proteins are not presented on the surface of infected cells until the cell is lysed and virus released. However, ADCP could increase the uptake of RV virions by immune cells, which could reduce viral load, facilitate antigen presentation, and stimulate the secretion of inflammatory mediators^31,32^. It is important to note that the ADCP assay in this study does not assess the functional reduction in infectious viral titers, which will require further investigation. It is possible that during the epitope bound by mAb HRV-18003 is preferentially exposed upon conjugation to beads, contributing to the observed effect.

An earlier study has shown that human polyclonal antibody responses target an epitope distinct from the one identified here, that is also on VP1 and similarly not exposed on the virion surface^33^. It would be interesting to test the ability of these antibodies to mediate ADCP, to investigate if they may contribute to protection through non-traditional mechanisms. If future studies indicate a potential protective role for these antibodies, ADCP may be an interesting mechanism to be explored in addition to neutralization for vaccine development against RV.

Importantly, Fc-mediated effector functions such as ADCP are substantially affected by the IgG subtype^34^. All mAbs used in this study (including the negative control), with the exception of HRV-18009 (IgG2b) were IgG2a – which have both been shown to mediate similar levels of Fc-receptor engagement. The observed differences were therefore likely mediated by the affinity and/or specificity of the antibody Fab-domain. The differences observed between mAbs HRV-18003 and HRV-18004 were most likely due to differences in affinity since they both bind to the same epitope and express the same Fc-domain. However, the differences observed compared to some of the strong binding RV-A15-specific mAbs could be epitope-specific. It has been previously shown for other viruses that Fc-mediated effector functions can be epitope-specific, which remains to be explored for RVs^35^.

In summary, we have discovered cross-reactive mAbs that bind a conserved epitope on VP1. Further, one cross-reactive mAb (HRV-18003) demonstrated a high level of ADCP activity *in vitro*, which may be a mechanism that could be explored for future vaccine development against RV.

## Methods

### Cells and viruses

HeLa H1 cells (ATCC CRL-1958) were grown in complete Dulbecco’s modified Eagle medium (DMEM; Life Technologies) supplemented with antibiotics (100 U/ml penicillin, 100 μg/ml streptomycin [Pen-Strep]; Gibco), and 10% fetal bovine serum (FBS; HyClone). Hybridoma cell culture was performed as previously described^36^. Briefly, SP2/0 mouse myeloma cells were cultured in complete DMEM supplemented with antibiotics (Pen-Step; cDMEM) prior to fusion with primary mouse splenocytes. Monoclonal hybridoma cells were initially grown in Clonacell-HY Medium E (Stemcell Technologies) and subsequently switched to serum-free hybridoma medium (Hybridoma-SFM; Life Technologies) for antibody production. RAW 264.7 macrophages (ATCC TIB-71) were cultured in RPMI 1640 containing 10% FBS. RVs 1A, 1B, 14, 15, 16, 49, 68, and 71 were purchased from ATCC and passaged in HeLa H1 cells. To create purified viruses, infected cell cultures were lysed by freeze/thawing and clarified supernatants harvested after low-speed centrifugation (at 3,000 g for 30 min at 4 °C) to remove cellular debris. Viruses were pelleted through a 30% sucrose cushion (30% sucrose in NTE buffer (100 mM NaCl, 10 mM Tris-HCl, 1 mM EDTA), pH 7.4) by ultracentrifugation (Beckman L7-65 ultracentrifuge with SW-28 rotor at 25,000 rpm for 5 h). Once all of the supernatant was aspirated, virus pellets were resuspended in phosphate-buffered saline (PBS; Life Technologies).

### Construction of plasmids

The polyprotein coding region of P1-2A (2.3 kb) and 3C viral protease (639 bp) of RVs A1A, A1B, B14, A16, A49, A68, and A71 were amplified using corresponding primers. The PCR products of P1-2A and 3C were cloned into a pCAGGS vector plasmid using In-Fusion cloning (Takara Bio; Fig. 1A). Positive clones were selected by colony PCR and the plasmids confirmed by nucleotide sequencing.

To generate recombinant capsid proteins (VP1-4) of RV-A15 and mutant VP1 of RV-A15 and RV-A68 wt and mutant VP1 (sequences of mutant VP1s are shown in Fig. 6B), the corresponding fragments were amplified by PCR. A C-terminal His_6_ tag was further added to the amplicons, as previously described^20,37^. The PCR products were cloned into the pQE30 expression vector (Qiagen, Hilden, Germany), and the constructs were screened by sequencing. Protein expression in *Escherichia coli* SG13009 (pREP4) bacteria containing the pQE30 recombinant vector was induced by addition of isopropyl-β-d-1-thiogalactopyranoside (IPTG) to a final concentration of 0.5 mM. Soluble recombinant protein was purified on Ni-nitrilotriacetic acid agarose columns using Ni-NTA Fast Start Kit (Qiagen, Hilden, Germany) under native conditions according to the manufacturer’s instructions. The purified proteins were verified by sodium dodecyl sulfate-polyacrylamide gel electrophoresis (SDS-PAGE) and confirmed with Western blot analysis using anti-His antibody (Qiagen, Hilden, Germany) (Supplementary Fig. 8). The concentrations of the purified VPs were determined by Bradford protein assay testing (Bio-Rad, Hercules, CA).

### Generation and screening of mAbs

Six- to eight-week-old female BALB/c mice (n = 2) were immunized with 75 μg DNA encoding P1-2A and 3C of different strains of RVs in pCAGGS plasmids (Fig. 1B). Injections were performed bilaterally with 50 μl per leg (total volume of 100 μl). Each animal received four vaccinations, 3 weeks apart, via intramuscular electroporation in the medial thigh using a TriGridDelivery System from Ichor Medical Systems. Mice were bled 4 weeks post each vaccination to monitor antibody responses against RVs (Supplementary Fig. 9). Three days prior to hybridoma fusion, mice were boosted with 50 μg of purified RV-A15 (Fig. 1B). The final boost followed a short interval before spleen harvest aims to boost B cells, but to elicited only limited *de novo* responses. B cell hybridomas were generated as previously described^36^. All animal procedures were approved by the Icahn School of Medicine at Mount Sinai Institutional Animal Care and Use Committee (IACUC) and performed in accordance with the guidelines set by the committee.

### Screening of hybridoma supernatants

Hybridoma supernatants were screened by ELISA (Enzyme-linked immunosorbent assay) for reactivity with multiple strains of RVs (purified, whole virus) including 1A, 1B, 14, 15, 16, 49, 68, and 71. All wells that had activity (defined as signal over background) against any virus were then isotyped using a Pierce rapid antibody isotyping kit (Life Technologies). The isotyped mAbs expressing IgG heavy-chain subclasses were selected for further expansion and purification, as previously described^38^. All mAbs, except for HRV-18009 (IgG2b) were IgG2a.

### Binding characterization (ELISA)

ELISAs were performed as previously described^39^. Briefly, Microtiter 96-well plates (Immulon 4HBX; Thermo Fisher Scientific) were coated with 5 μg/ml purified RVs or 2 μg/well of recombinant VP1-VP4 proteins (50 µl/well) from RV-A15 or mutant or wt. of RV-A15 or RV-A68 diluted in coating solution (KPL, Gaithersburg, MD), and were incubated at 4 °C overnight. Plates were washed 3x with phosphate-buffered saline (PBS; Gibco) containing 0.1% Tween 20 (PBS-T; Fisher) and then blocked with PBS-T containing 3% goat serum (Life Technologies, Inc.) and 0.5% milk powder (blocking solution) for 1 h. Three-fold antibody dilutions were performed in blocking solution at a concentration of 30 μg/mL in a final well volume of 100 μL. After 2 h plates were washed 3x with PBS-T, followed by 1 h staining with goat anti-mouse IgG Fc-chain horse radish peroxidase secondary antibody (abcam, ab98717) diluted 1:10,000 in blocking solution at a final well volume of 50 μl. Plates were washed 4x with PBS-T, followed by development with SigmaFast o-phenylenediamine dihydrochloride (OPD; Sigma). The reaction was stopped with 3 M hydrochloric acid. Binding of antibodies to the antigens was detected at an optical density (OD) of 490 nm using by a Synergy H1 hybrid multimode microplate reader (BioTek).

### Microneutralization (MN) assay

To evaluate the functionality of the mAbs against different types of RVs, a MN assay based on measuring viability of HeLa cells using 3-(4,5-dimethylthiazol-2-yl)-5-(3-carboxymethoxyphenyl)-2-(4-sulfophenyl)-2H-tetrazolium (MTS) was used^40^. Using MTS over crystal violet solution allowed for a more incremental quantification using OD measurements. First, TCID_50_s (50% tissue culture infectious dose) of each RV strain were measured on Hela cells. The cells were seeded at the concentration of 3.5*10^5^ /mL 24 hours prior to the assay in 96-well plates. At the time of the assay, the viruses were serially diluted (half-log_10_ dilutions) in Hela infectious media (10X MEM supplemented with 100 U/ml penicillin, 100 μg/ml streptomycin [Pen-Strep]; Gibco), and 10% fetal bovine serum (FBS; HyClone), 1% Non-essential amino acids (NEAA; Gibco), and 1% 3 M MgCl2), added to the cells (100 μl per well), and incubated at 33 °C for 1 h. After incubation, the cells were washed and overlaid with 100 μl of infectious media and incubated at 33 °C for 72 hours. After an incubation period, virus replication was visualized by measuring the viability of cells with an MTS assay kit (abcam, ab197010), as described previously^40^. All the tests were done in triplicate. TCID_50_/ml was calculated according to the Reed-Muench method. To perform the MN assay, 100× TCID_50_ (50% tissue culture infectious dose) of each RV strain were mixed with 2-fold serial dilutions of each mAb (starting at the concentration of 120 μg/mL, 2-fold dilution) in HeLa infectious media and incubated to allow binding of the antibodies to the viruses for 1 h at room temperature (RT). The virus-mAb mixture was then added onto HeLa cells and incubated at 33 °C for 1 h. After the incubation period, the virus-mAb mixture was removed and replaced with diluted mAbs at the previous concentration. After an incubation period of 72 h, virus replication was measuring as described above using an MTS assay kit. Plates were read at an OD of 490 nm and the IC_50_ of each mAb for each virus was calculated in GraphPad Prism version 7.0 (GraphPad Software, San Diego, CA).

### Antibody-dependent cellular phagocytosis (ADCP) assay

The assay was performed according to previously described methods^41,42^.To measure ADCP, viruses (RV-A15, RV-A1A or RV-A16) were biotinylated using an EZ-Link NHS-PEG4-Biotin kit (Thermo Fisher Scientific) and conjugated to FluoSpheres NeutrAvidin-Labeled Microspheres beads (0.2 μm, yellow-green, Life Technologies) according to manufacturer’s instructions. Antibodies (2-fold diluted from 200 μg/ml to 25 μg/ml) were incubated with beads for 2 h at 37 °C. RAW cells were added at a concentration of 1.0 × 10^5^ cells/well and incubated for 1 h at 37 °C. Cells were analyzed by flow cytometry on a Gallios flow cytometer (Beckman Coulter), and fold-increase compared to cells with RV-conjugated beads only was calculated based on the gating strategy shown in Supplementary Fig. 3. All mAb samples were tested in duplicate.

### Western blotting

Binding of mAbs to RV protein was analyzed on SDS-PAGE (4–20% polyacrylamide; Mini Protean TGX gels; Bio-Rad) under reducing conditions. Briefly, the protein samples were boiled for 5 minutes at 100 °C in 2× Laemmli sample buffer (Bio-Rad) containing 5% β-mercaptoethanol and subjected to SDS-PAGE electrophoresis, and then transferred onto nitrocellulose membranes using the iBlot 2 dry blot system (Thermo Fisher) at 20 V for 7 min. Following blocking with 3% skim milk for 2 h at RT, the membrane was incubated with primary antibody overnight at 4 °C. After three washes with 1×PBST buffer (1× phosphate-buffered saline, 0.05% Tween 20), HRP-conjugated goat anti-mouse IgG (abcam) was added to the membrane and incubated at RT for 1 h. The positive bands were visualized by electrochemiluminescence (ECL) reagent (Life Technologies) and developed using1-Step Ultra TMB-Blotting Solution (Life Technologies).

### Generation of RV escape mutants against neutralizing mAbs

Monoclonal antibody escape mutants of RV-A15 were generated as previously described with some modifications^36,43^. Initially, HeLa cells in 6-well tissue culture plates (Sigma) were infected with RV-A15 at a multiplicity of infection (MOI) of 1 in the presence of 1×IC_50_ of mAb (performed in duplicate for each mAb) in Minimum Essential Medium (MEM; Life Technologies). After incubation for 72 h at 33 °C, 100 μl of supernatant was collected and used to directly inoculate a fresh monolayer of HeLa cells in the presence of a 2-fold increase in the mAb concentration. This process was repeated for seven passages, until the final concentration of mAb was 128×IC_50_. Throughout serial passaging, successful infection was confirmed by the presence of cytopathic effect. To control for mutants obtained from passaging alone, the virus was also passaged in the presence of an irrelevant mouse mAb, and without mAb. Escape mutants were plaque purified once serial passaging was completed to create stocks for sequencing.

### Epitope mapping of HRV-18003 using a peptide microarray

The epitope of mAb HRV-18003 in the VP1 capsid protein of RV-A15 was mapped using peptide microarray technology performed by PEPperPRINT (PEPperCHIP Platform Technology, Heidelberg, Germany). Briefly, the sequence of VP1 capsid protein of RV-A15 was translated into 7, 10 and 13 amino acid peptides with a peptide-peptide overlap of 6, 9 and 12 amino acids, respectively. The resulting peptide microarrays contained 885 different cyclic peptides printed in duplicate (1,770 peptide spots), and were framed by additional HA (YPYDVPDYA) control peptides. Blocking, washing, and incubation procedures were performed using Rockland blocking buffer MB-070 (VWR International Frankfurt, DE) (PBS plus 0.05% Tween 20, PBS-Tween plus 10% Rockland blocking buffer, respectively). Mouse mAb HRV-18003 was incubated at a concentration of 0.1 µg/ml in incubation buffer followed by staining with the secondary antibody (Goat anti-mouse IgG-DyLight680, New England Biolabs, Frankfurt, DE).

## Supplementary information

Supplementary information.

## Data Availability

The datasets generated during the current study are available from the corresponding author upon request.
