# Supplementary material for: A cross-reactive mouse monoclonal antibody against rhinovirus mediates phagocytosis in vitro
Source: Sci Rep. 2020 Jun 16;10:9750. doi: 10.1038/s41598-020-66600-x (PMC7297972; doi:10.1038/s41598-020-66600-x)

Supplementary data

A cross-reactive mouse monoclonal antibody against rhinovirus mediates phagocytosis *in vitro*

Mohammad Amin Behzadi[1], Angela Choi[1,2,3], James Duehr[1,4], Roya Feyznezhad[5], Chitra Upadhyay[5], Michael Schotsaert[1,2], Peter Palese[1,5], Raffael Nachbagauer[1,*]

[1] Department of Microbiology, Icahn School of Medicine at Mount Sinai, New York, NY, USA

[2] Global Health and Emerging Pathogens Institute, Division of Infectious Diseases, Icahn School of Medicine at Mount Sinai, New York, NY, USA

[3]Graduate School of Biomedical Sciences, Icahn School of Medicine at Mount Sinai, New York, NY, USA

[4] Current affiliation: University of Pittsburgh School of Medicine, Pittsburgh, PA, USA

[5] Department of Medicine, Division of Infectious Diseases, Icahn School of Medicine at Mount Sinai, New York, NY, USA

***** raffael.nachbagauer@mssm.edu

Supplementary Figure 1. Reactivity of isolated mAbs against different subtypes of HRV. Individual ELISA data shown here have been summarized as a heat map in Fig. 2B.

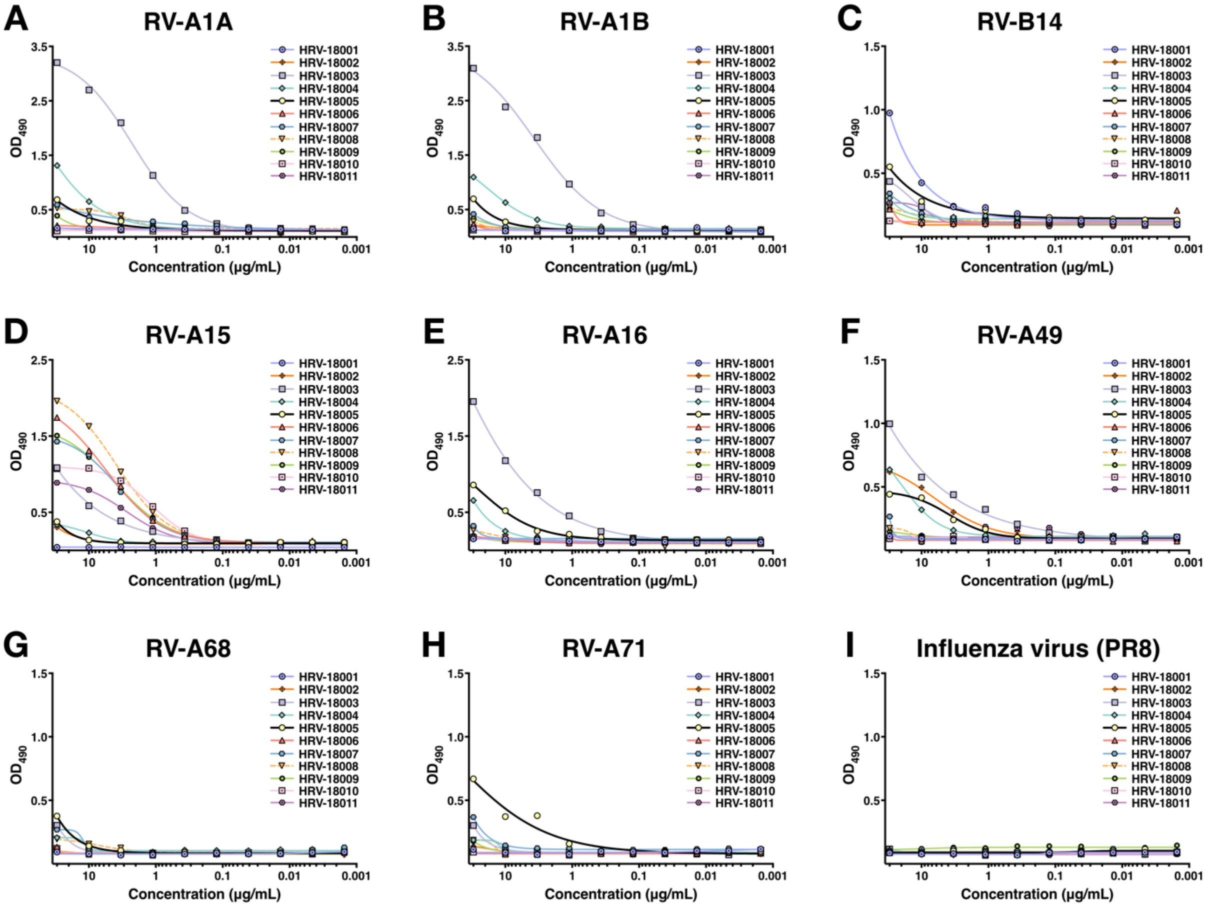

Supplementary Figure 2. Neutralizing activity of mAbs against different subtypes HRVs. Microneutralization assays shown here were used to create the heat map in Fig. 3A.

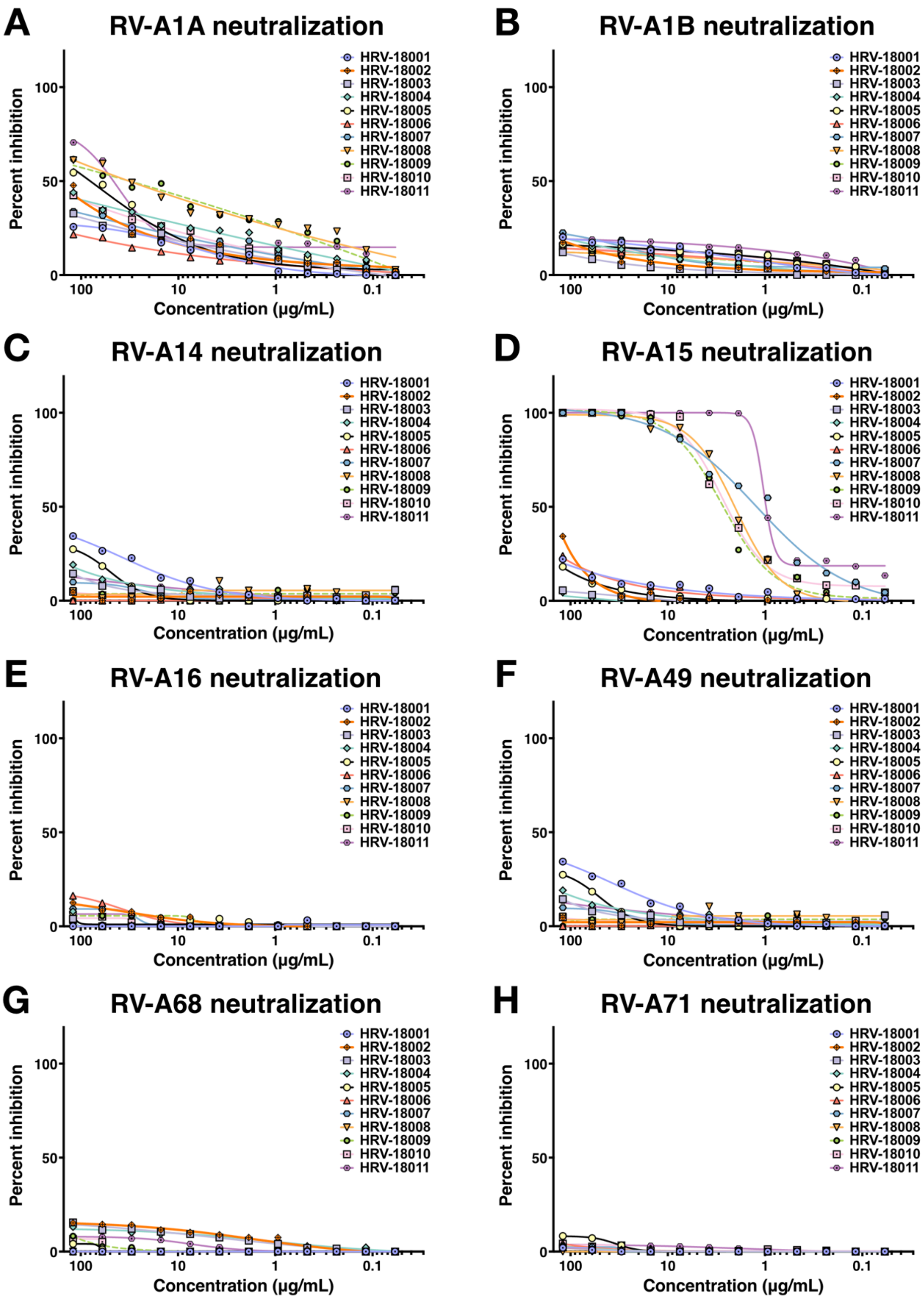

Supplementary Figure 3. Flow cytometry gating strategy. Representative examples of (**A**) cells and beads only, (**B**) a negative isotype control, and (**C**) mAb HRV-18003 with the gating strategy used for the ADCP assay. A flow cytometry-based assay was developed to measure mAb ADCP activity. For analysis, cells were first gated on single cells [A]. Next, all cells were gated [B], and within that population, bead-positive cells were detected in gate [C].

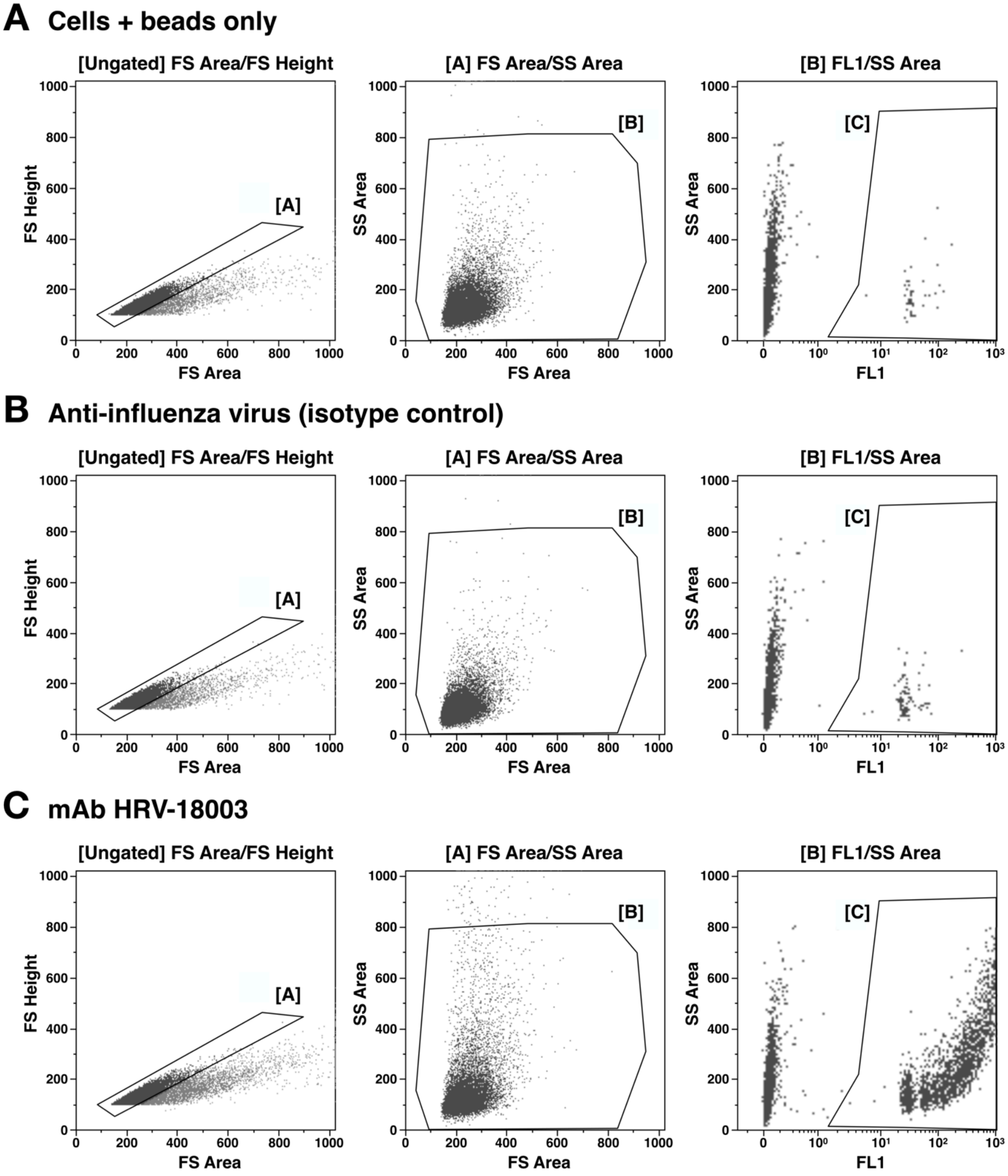

Supplementary Figure 4. Antibody dependent cellular phagocytosis (ADCP) activity of mAbs against additional RVs. Cross-reactive mAbs HRV-18003 and HRV-18004 were compared to HRV-18008. An anti-influenza virus mAb (2B9; IgG2a) was used as a negative control. Values are depicted as fold-induction over cells and virus-conjugated beads without antibody. All assays were performed in duplicate. Bars show the mean and error bars indicate the standard error of the mean. The results are consistent with ADCP results against RV-A15 and the ELISA binding data, with HRV-18008 showing low binding to RV-A1A by ELISA and ADCP activity, while not showing activity in either assay against RV-A16. **A)** ADCP assay using RV-A1A. **B)** ADCP assay using RV-A16.

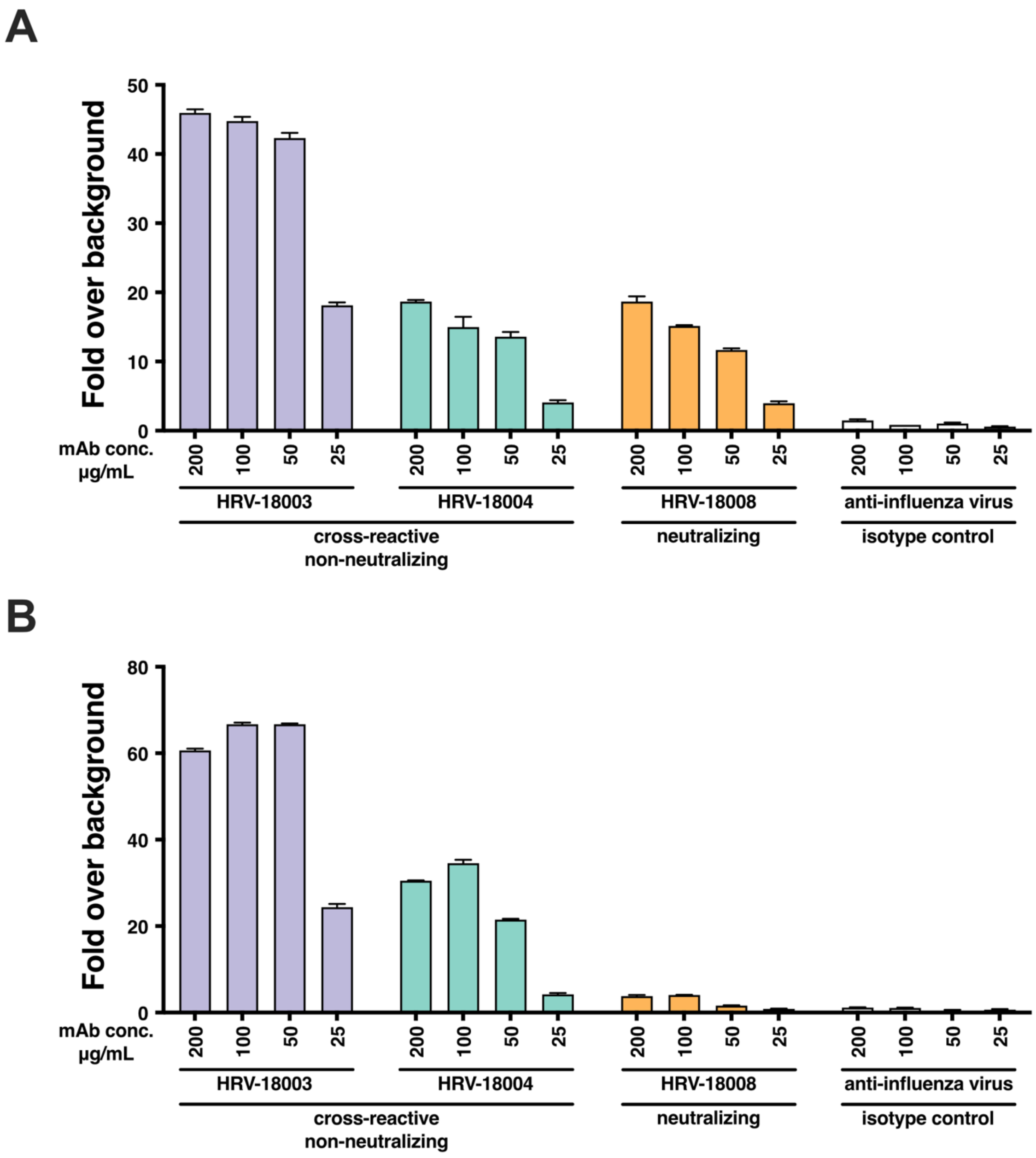

Supplementary Figure 5. Neutralizing activity of the mAbs against escape mutants. Microneutralization assays were performed using the five HRV15 escape mutants (plus wildtype virus) with each of the five neutralizing mAbs.

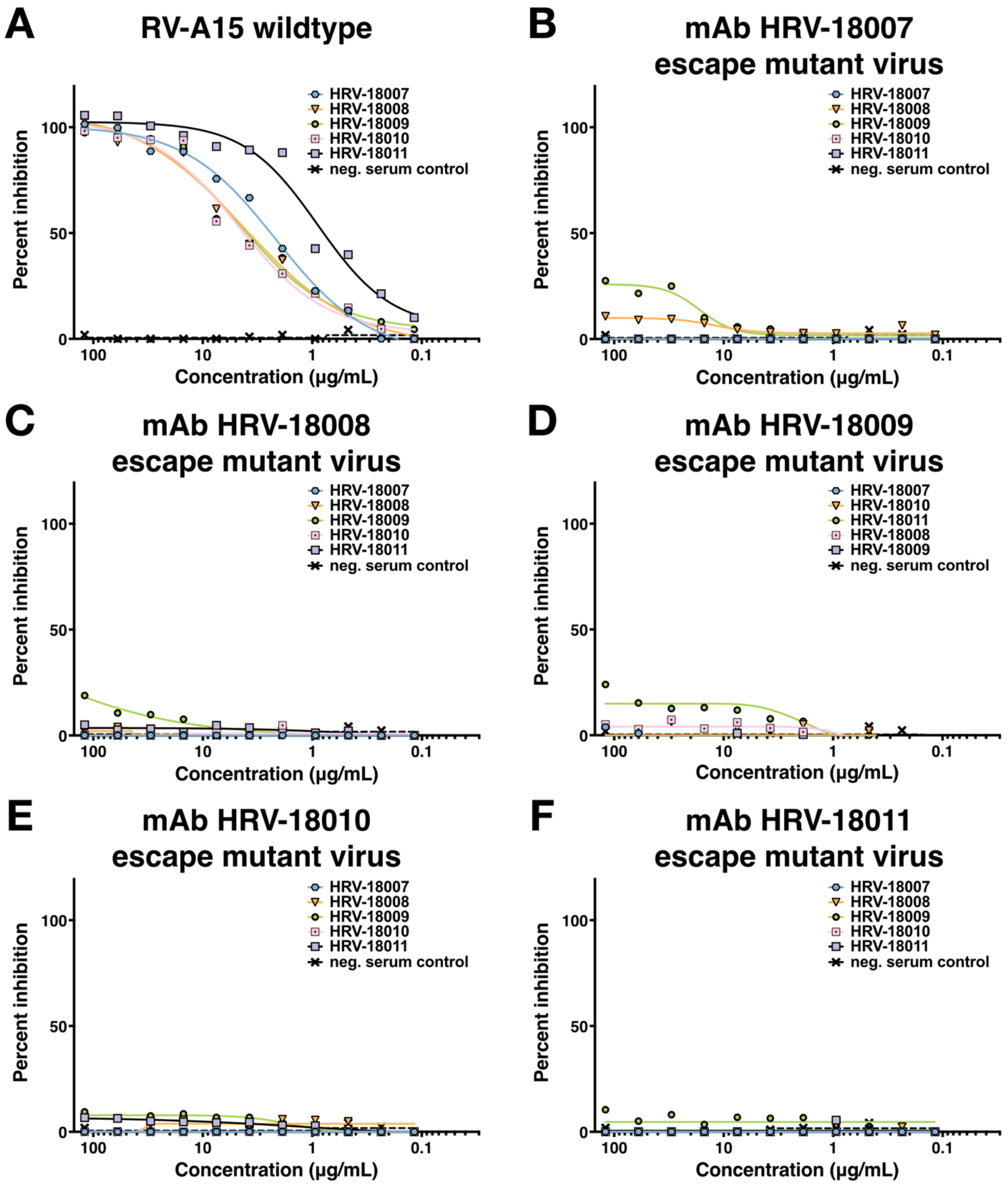

Supplementary Figure 6. Western blot analysis on multiple subtypes of HRVs using cross-reactive mAbs. Western blots were performed with 5 μg of different HRVs (lanes 1-8) and PR8 influenza virus (lane 9) (negative control). Membranes were stained with mAb HRV-18003, or mAb HRV-18004 followed by secondary staining with anti-mouse IgG coupled to HRP. **A**) Staining with mAb HRV-18003. **B**) Staining with mAb HRV-18004.

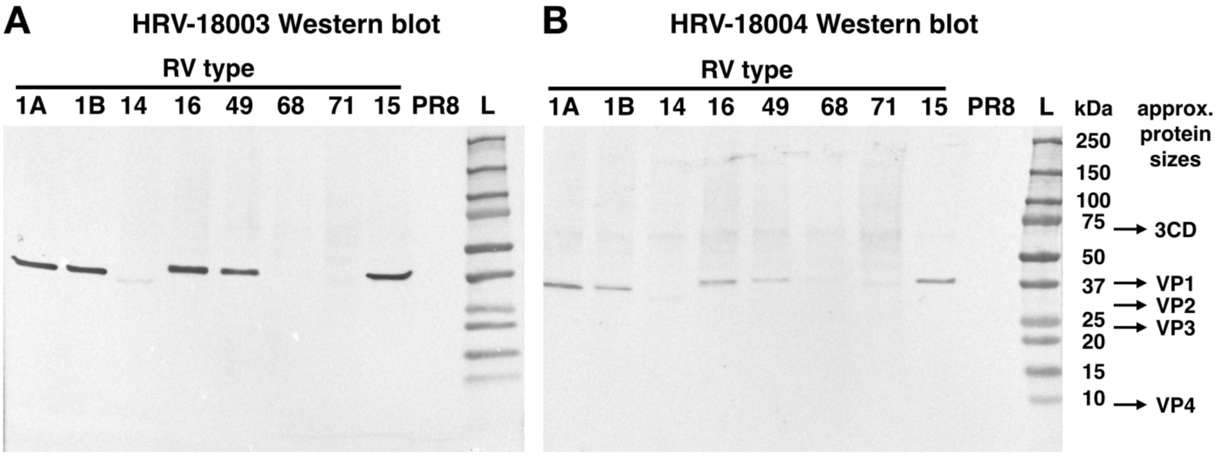

Supplementary Figure 7. Binding epitope of broadly cross-reactive mAb HRV-18003. The binding site of mAb HRV-18003 epitope was visualized on a crystal structure of the HRV1A (PDB accession no. 1R1A). Amino acid sequences of the presumed epitope are highlighted in blue. The relevant changed amino acid of the epitope is not exposed on the virion surface. Data visualized in PyMoL (Schrödinger, version 2.3.2, www.pymol.org).

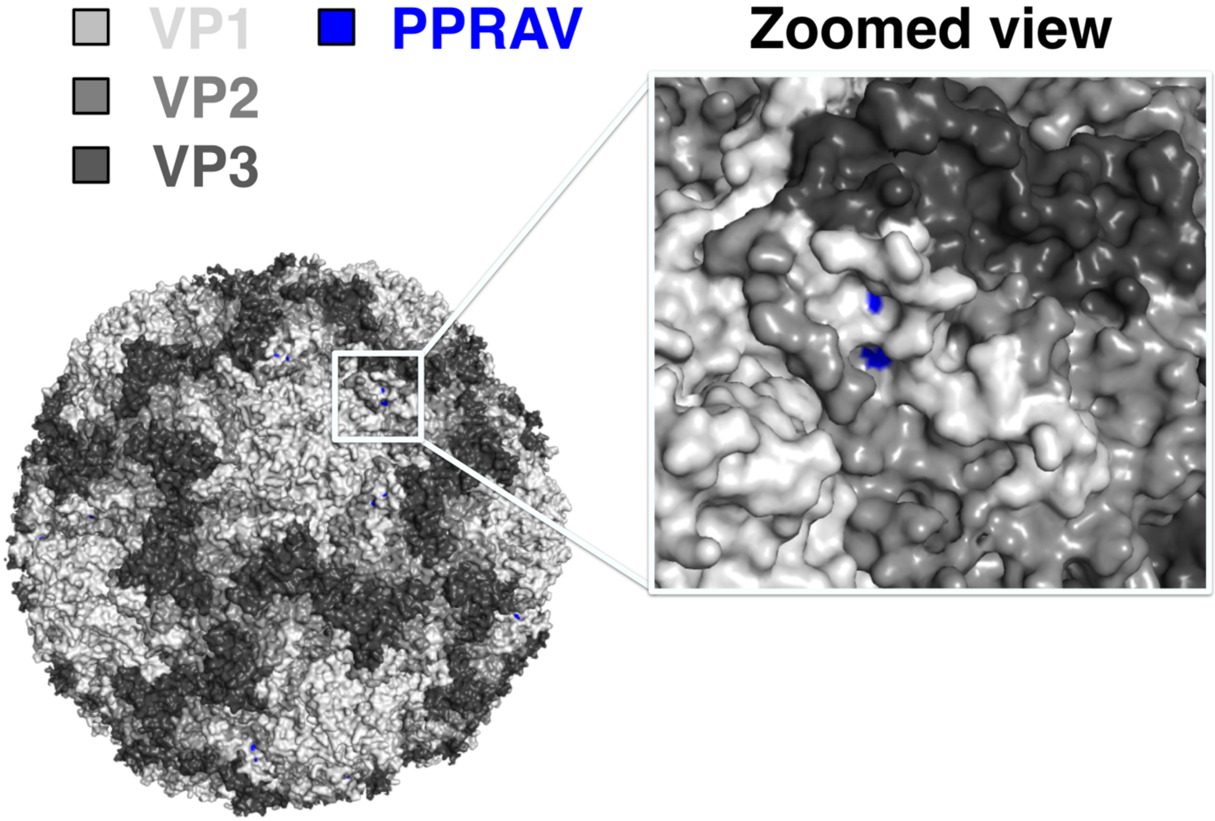

Supplementary Figure 8. RV-A15 capsid proteins Western blot. Recombinant capsid proteins (VP1, VP2, VP3 and VP4) produced in *E. coli* were tested in a Western blot using an HRP-labeled anti-His antibody for detection. The sizes for the proteins are as expected with the exception of VP4, which was detected at a larger size than expected. This could indicate potential multimerization of the VP4 protein.

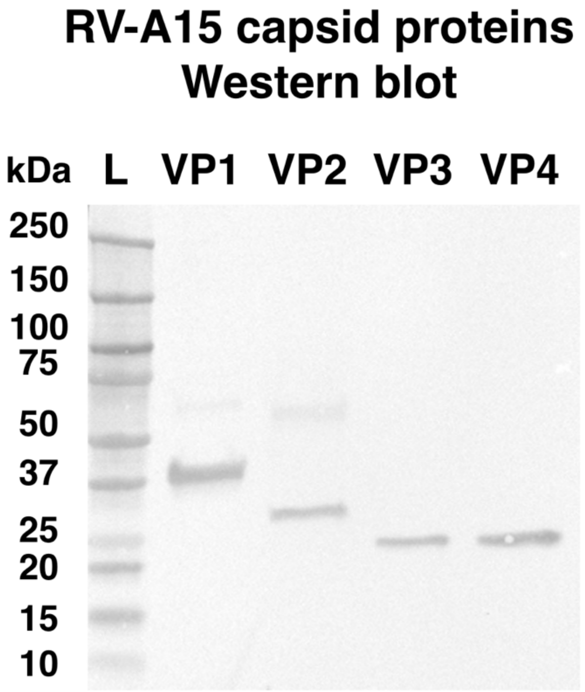

Supplementary Figure 9. Binding of post-vaccination mouse sera by ELISA. Mice were bled 4 weeks post each vaccination and sera were tested for binding against RVs A1A **(A)**, A1B **(B)**, B14 **(C)** and A16 **(D)**. Naïve mouse sera were included as negative controls. Binding of mouse sera to whole virus increased after repeated vaccination.

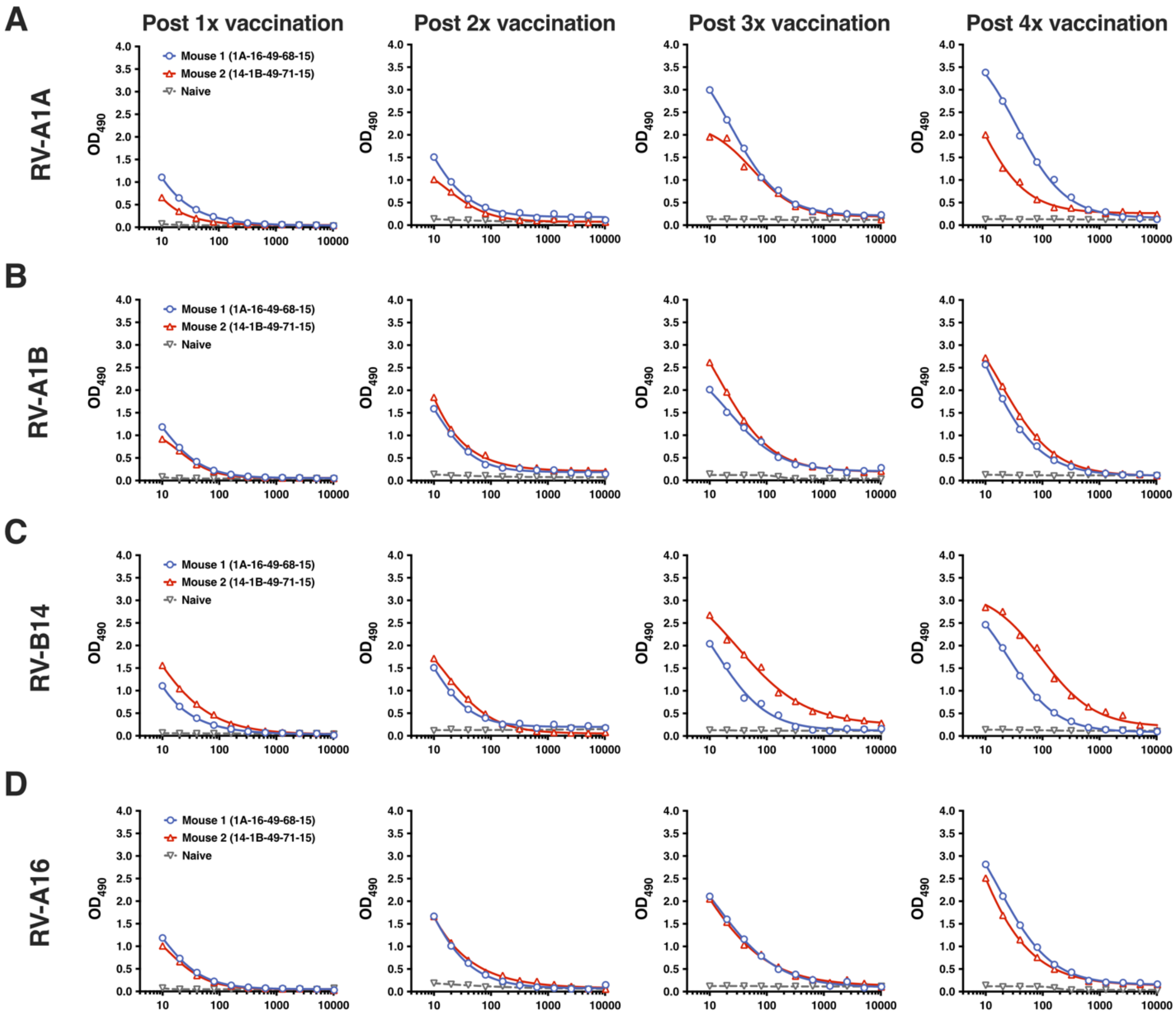

Supplement: Supplementary file 1 — Supplementary information. [file 41598_2020_66600_MOESM1_ESM.pdf]
